# Glomerular Diseases in Diabetic Patients: Implications for Diagnosis and Management

**DOI:** 10.3390/jcm10091855

**Published:** 2021-04-24

**Authors:** Nestor Oliva-Damaso, José María Mora-Gutiérrez, Andrew S. Bomback

**Affiliations:** 1 Department of Medicine, Division of Nephrology, Hospital Costa del Sol, 29603 Marbella, Malaga, Spain; nestorod@hotmail.com; 2 Department of Medicine, Division of Nephrology, Clínica Universidad de Navarra, 31008 Pamplona, Navarra, Spain; jmora@unav.es; 3 Department of Medicine, Division of Nephrology, Columbia University Irving Medical Center, New York, NY 10032, USA

**Keywords:** diabetes mellitus, glomerulonephritis, nondiabetic renal disease, nondiabetic kidney disease, focal segmental glomerulosclerosis, obesity-related glomerulopathy, IgA nephropathy

## Abstract

The prevalence of diabetes continues to rise worldwide. In addition to rising rates of diabetic kidney disease, we are also seeing a parallel rise in nondiabetic kidney disease among patients with diabetes. These nondiabetic lesions include focal segmental glomerulosclerosis, IgA nephropathy, membranous nephropathy, and other glomerular diseases. The management of diabetic kidney disease is rapidly evolving to include, beyond glycemic control and renin angiotensin inhibition, the use of sodium-glucose cotransporter 2 (SGLT2) inhibitors and mineralocorticoid receptor antagonists. These and other new treatment strategies should be applicable to managing glomerular disease in diabetic patients to reduce toxicities associated with immunosuppression and, in particular, corticosteroids. The prevalence of glomerular disease in diabetic patients is underappreciated. Diagnosis and appropriately treating these diseases remain an important avenue to modify kidney outcomes in diabetic patients.

## 1. Introduction

Diabetic kidney disease (DKD) is the leading cause of disability-adjusted life years (DALYs) in chronic kidney disease (CKD), accounting for 30.7% of the total CKD DALYs [1]. The prevalence of diabetes mellitus (DM) among United States adults is 12.2% of the general population, and CKD is frequent in DM, with 36% of diabetic adults manifesting some degree of CKD [2]. Fortunately, recent developments in therapeutics suggest new approaches to improve outcomes in DKD [3], including the use of sodium-glucose cotransporter 2 (SGLT2) inhibitors, glucagon-like peptide 1 (GLP-1) receptor agonists, and third generation mineralocorticoid receptor antagonists.

Diabetic kidney disease is defined as having reduced kidney function or albuminuria in patients with DM [4]. This term is a clinical diagnosis, confirmed but not requiring a kidney biopsy, which may involve diverse causes including nondiabetic kidney disease (NDKD) such as hypertensive nephrosclerosis, unresolved acute kidney injury (AKI), obesity-related glomerulopathy, and a myriad of other glomerular lesions. Diabetic glomerulosclerosis is a diagnosis that refers to specific pathologic structural changes and functional changes seen in the kidney biopsies of patients with DM that results from the direct effects of DM on the kidneys [4]. NDKD, particularly glomerular lesions not attributed to DM, remains to be an underappreciated, underexplored, and an increasingly recognized phenomenon [5].

Two large-scale, retrospective examinations of kidney biopsies of patients who had been diagnosed with diabetes revealed that the majority of patients (63–72.5%) had NDKD lesions either alone or alongside diabetic glomerulosclerosis [6,7]. In these two studies, focal segmental glomerulosclerosis (FSGS) was the most common finding in the NDKD alone group (just over 20%), followed by hypertensive nephrosclerosis, acute tubular necrosis, IgA nephropathy (IgAN), and membranous nephropathy (MN). European cohorts have reported hypertensive nephrosclerosis and IgAN as the most common causes of NDKD [8], while Chinese studies have described MN and IgAN as being more prevalent [9].

The indication for a kidney biopsy in diabetic patients, usually prompted by an atypical course of kidney disease or clinical suspicion of NDKD, can differ across centers and limits these retrospective analyses. Prospective analyses with clearly defined indications for kidney biopsies, in which all type 2 diabetes (T2D) patients with proteinuria greater than 1 g per day were referred for biopsy, showed an important but lower prevalence of NDKD alone or alongside DKD (33%) [10,11]. Thus, the “true” prevalence of NDKD is unknown. However, the aforementioned data show a significant number of patients with potentially treatable, reversible NDKD lesions. The elevated risk of FSGS in African Americans and IgAN in Asians has led to the discovery of race-determined genetic risk variants [12,13]. This may influence the prevalence of CKD in different regions, including among diabetic patients. In other words, NDKD is common in patients with diabetes [14], while population background influences the heterogeneity of NDKD [15].

The review of native kidney biopsy findings in diabetics performed at the Columbia Renal Pathology Laboratory, in 2011, [6] revealed that one of four native kidney biopsies was performed in a patient with diabetes. Diabetic patients whose biopsies revealed NDKD alone had a shorter course of DM and subnephrotic proteinuria, while long-term DM was a predictor of diabetic glomerulosclerosis alone. Most kidney biopsies performed in patients with diabetes occur in advanced stages of kidney disease. In this cohort, the median estimated glomerular filtration rate (eGFR) was 29 mL/min per 1.73 m^2^ with nephrotic range proteinuria at the time of biopsy [6]. In the last decade, a marked increase in the histological diagnosis of diabetic glomerulosclerosis (from 5.5% to 19.1%) has been reported [16]. This reflects the increasing incidence of the disease as a consequence of the rising incidence of DM [17]. It also denotes an underlying tendency to biopsy older patients or to look for NDKD among diabetic patients [16].

## 2. Obesity-Related Glomerulopathy and Secondary FSGS in Diabetics

The driving force behind the increase in diabetes prevalence is the global pandemic of obesity [18]. Obesity (body mass index >30 kg/m^2^) is an independent risk factor for kidney disease progression [19], especially visceral obesity [20]. Systemic conditions such as hypertension and obesity are risk factors for kidney disease progression, particularly in DKD [21]. In T2D, systemic hypertension and obesity contribute to glomerular hyperfiltration due to high transmitted systemic blood pressure and glomerular enlargement [22,23]. Obesity leads to a secondary form of FSGS, termed obesity-related glomerulopathy (ORG), independent of diabetic status [24]. The modern spectrum of kidney biopsy findings, in patients with morbid obesity, highlights that diabetic glomerulosclerosis is the most common associated glomerular pathology finding. Approximately 40% of patients with obesity can have features of DKD with mesangial expansion, glomerular basement membrane thickening, and/or nodular glomerulosclerosis [25]. Among 3263 native kidney biopsies at Columbia University, in 2017, 8% were of morbidly obese patients, with 47% of these patients, in turn, carrying a diagnosis of DM [25]. ORG and DKD share clinical and pathogenic features, such as activation of the renin-angiotensin-aldosterone system (RAAS), sodium retention, activation of the sympathetic nervous system, and increased intra-glomerular capillary pressure creating hyperfiltration, podocyte injury, and adaptive (i.e., secondary) FSGS lesions [26,27]. Primary FSGS with diffuse podocyte foot process enfacement in diabetic patients is an extremely rare condition (<1%) [6].

The RAAS inhibitors are pivotal in the treatment and control of DKD, as they are associated with a reduction in the progression of disease with regards to both creatinine/eGFR and proteinuria [3,28]. These agents are also crucial in the treatment of patients with secondary FSGS [29]. RAAS antagonism therapy directly addresses the hemodynamic alterations in adaptive FSGS. Of note, endothelin type A receptor antagonists (ERAs) could provide additive protective effects to RAAS inhibitors for reducing proteinuria in patients with diabetic nephropathy (DN) [30,31]. This was evaluated in patients with FSGS in the DUET study, a phase 2 study with 109 patients, where sparsentan (a dual endothelin type A and angiotensin II type 1 receptor antagonist) showed more potent reduction in proteinuria after 8 weeks of treatment as compared with irbesartan. This trial only included well-controlled T2D participants and did not stratify subgroups by diabetes status [32]. The ongoing DUPLEX trial is a phase 3 study designed to assess the impact of sparsentan on eGFR slope and proteinuria in 300 participants with FSGS confirmed by biopsy. The DUPLEX study excluded patients with type 1 diabetes, uncontrolled T2D, or non-fasting blood glucose >180 mg/dL at screening [33].

Mineralocorticoid receptor antagonism (MRA) plays a role in the control of glomerular diseases by reducing proteinuria and attenuating progressive renal disease in CKD patients [34]. A low dose of spironolactone, added to angiotensin-converting enzyme inhibitor (ACEi) therapy, reduced blood pressure and urinary albumin excretion in obese hypertensive subjects [35]. The role of MRAs, added on top of conventional RAAS inhibition, has been shown to improve outcomes in DKD. This was recently demonstrated in a large sample of patients using third generation MRA, finerenone, in the FIDELIO-DKD study [36]. In patients with CKD and T2D, treatment with finerenone resulted in patients having a lower risks of CKD progression and cardiovascular events as compared with the placebo. The addition of MRAs to ACE inhibitors or ARBs has been shown, in both diabetic and nondiabetic subjects, to reduce proteinuria by up to 50%, independent of blood pressure reduction [37,38]. The benefits of MRA add-on therapy to ACE inhibitor or ARB should extend to DM patients with proteinuric NDKD (e.g., IgA nephropathy, MN, and FSGS).

The recent multicentre DAPA-CKD trial [39] with 4304 participants analyzed the effect of dapagliflozin on sustained decline in eGFR of at least 50%, end-stage kidney disease, or kidney-related or cardiovascular death. During this study, 115 patients with FSGS lesions confirmed by kidney biopsy were included [40], although subgroup analyses of primary and secondary forms are not yet available. From these 115 patients with FSGS, 22 patients had T2D [41], notably a greater sample than in the DUET study. It is tempting to speculate that patients with FSGS lesions could benefit from dapagliflozin in terms of CKD progression [40], but full subgroup analysis of primary and secondary outcomes of patients with FSGS has still not been published. DAPA-CKD should provide exciting results backed by an important sample size and long term follow up. Other shorter and underpowered studies have failed to demonstrate this possible benefit of SGLT2 inhibitors in FSGS outcomes. In the DIAMOND study [42], with only 11 FSGS patients without diabetes, dapagliflozin did not affect proteinuria after six weeks of treatment. In another pilot study which included ten patients with FSGS and evaluated the effects of 8 weeks of dapagliflozin on GFR and proteinuria, dapagliflozin failed to demonstrate additional effects on body weight, proteinuria, or measured GFR [43]. A phase 2 randomized, double-blind study by Bays et al. enrolled 376 overweight and obese nondiabetic patients (with no kidney biopsy performed) to evaluate the effects of canagliflozin on body weight [44]. Even though there was significant reduction in body weight, no effect on proteinuria was observed.

The effect of glucagon-like peptide 1 (GLP-1) receptor agonists on ORG or FSGS is limited to case reports. Nevertheless, in the absence of kidney pathology data, GLP-1 receptor agonists have demonstrated the ability to slow diabetic kidney disease and reduce albuminuria with an important loss of body mass index (BMI) [3]. In patients with decreased kidney function and severe obesity, significantly reduced BMI after bariatric surgery was associated with improvement of eGFR (measured by cystatin C) at three years follow-up [45].

## 3. IgA Nephropathy in Diabetics

The DAPA-CKD trial [39] included 270 patients with IgAN (38 with concomitant T2D) [40] and can be considered to be the largest IgAN trial so far, with the potential to provide robust evidence supporting the role of SGLT2 inhibitors in the progression of CKD in IgAN patients [41]. A full subgroup analysis of IgAN patients in the DAPA-CKD trial has recently been published [46]. Among 270 participants with IgAN (254 with a confirmatory biopsy), 137 patients were randomized to dapagliflozin and 133 patients to a placebo with a median follow-up of 2.1 years. The mean age was 51.2 years, with a mean eGFR of 43.8 mL/min/1.73 m^2^; the median UACR was 900 mg/g (25th–75th percentile range 540–1515) and 14.1% had type 2 diabetes. The mean rates of eGFR decline with dapagliflozin and placebo were −3.5 and −4.7 mL/min/1.73m^2^/year, respectively. Dapagliflozin reduced the UACR by 26% relative to a placebo. Therefore, dapagliflozin reduced the risk of CKD progression when added to ACEi/ARB therapy with a favorable safety profile. This effect was consistent in patients with and without diabetes. This reveals an additive effect of SGLT2 inhibitors to the traditional treatment of this glomerular disease with RAAS inhibitors [41,46,47]. This dual, non-immunosuppressive approach could reduce the use of glucocorticoids in a specific group of patients with IgAN. Steroids carry a high morbidity profile in IgAN, as exemplified in the TESTING and STOP-IgAN trials [48,49], and these adverse effects are even worse in diabetic populations. The importance of these data lies in the prognostic implications of proteinuria reduction with RAAS blockade in IgAN [47], and future trials may need to include a background therapy of this dual kidney hemodynamic control of RAAS/SGLT2 inhibitors to control proteinuria and avoid eGFR decline in IgAN.

## 4. Other Glomerular Lesions

Little evidence exists to guide management of primary glomerular diseases in diabetic patients due to underrepresentation in clinical trials of glomerular diseases. The DAPA-CKD trial included 43 patients with MN (10 with concomitant T2D) and 11 patients with minimal change disease (2 patients with T2D) [39,40]. The MENTOR trial [50], a randomized controlled trial that found rituximab to be noninferior to cyclosporine in treating patients with MN, excluded patients with diabetes. Of note, the rituximab group was a steroid sparing immunosuppressive treatment that may be applicable to diabetics trying to avoid steroid toxicities such as worsening of hyperglycemia or weight gain.

**Figure 1:**
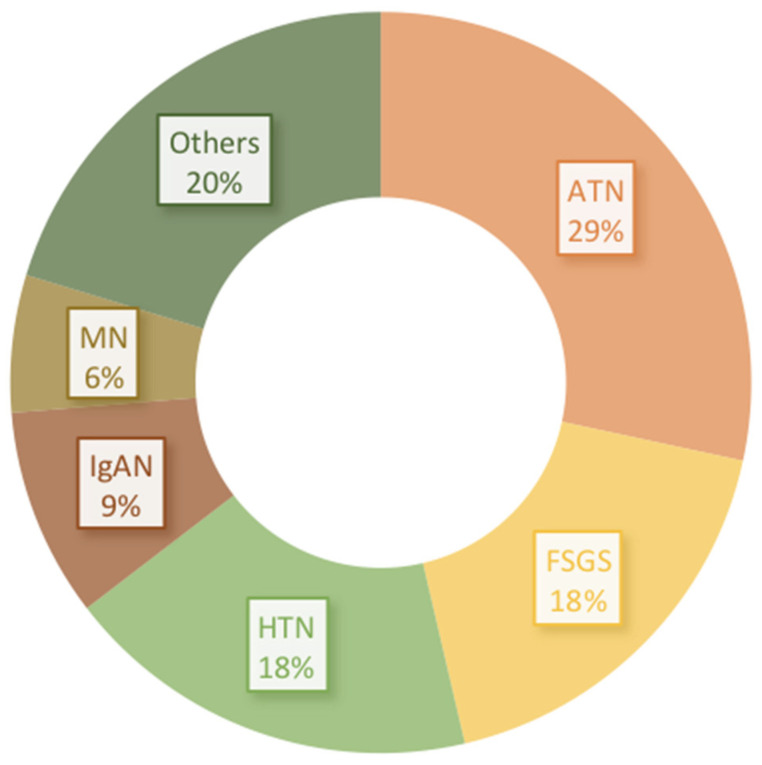
Kidney biopsy findings in a diabetic patient study from the Columbia Renal Pathology Laboratory, in 2011 [6]. Global frequencies including nondiabetic kidney disease (NDKD) alone and NDKD associated with diabetic nephropathy (DN). Focal segmental glomerulosclerosis (FSGS), hypertensive nephrosclerosis (HTN), acute tubular necrosis (ATN), IgA nephropathy (IgAN), membranous nephropathy (MN), and others.

Other coincident types of glomerular lesions in diabetic patients are possible but rare. The kidney biopsies findings in a diabetic patient study from the Columbia Renal Pathology Laboratory, in 2011, [6] showed that, in those patients with NDKD alone, FSGS (22%), hypertensive nephrosclerosis (18%), acute tubular necrosis (ATN) (17%), IgAN (11%), MN (8%), and pauci-immune glomerulonephritis (7%) comprised 80% of diagnoses as compared with ATN (43%), hypertensive nephrosclerosis (19%), FSGS (13%), and IgAN (7%) for NDKD with coexistent DKD. NDKD alone and associated with DN frequencies [6] are both shown in Figure 1.

## 5. When Should We Suspect Nondiabetic Kidney Disease (NDKD) and Biopsy?

A kidney biopsy in diabetics has diagnostic and prognostic implications. When NDKD is suspected, a kidney biopsy should be performed, as glomerular diseases may benefit from a different therapeutic approach than the standard of care for DKD alone. Lesions of NDKD likely contribute an important percentage of end-stage kidney disease (ESKD) in diabetics [51]. Classic indications for kidney biopsy in diabetics, such as absence of retinopathy, diabetes duration of less than 5 years, and microhematuria have been validated in type 1 diabetics, where importantly NDKD is rare (only 2–3%). The evidence in type 2 DM is largely retrospective [51,52].

**Figure 2:**
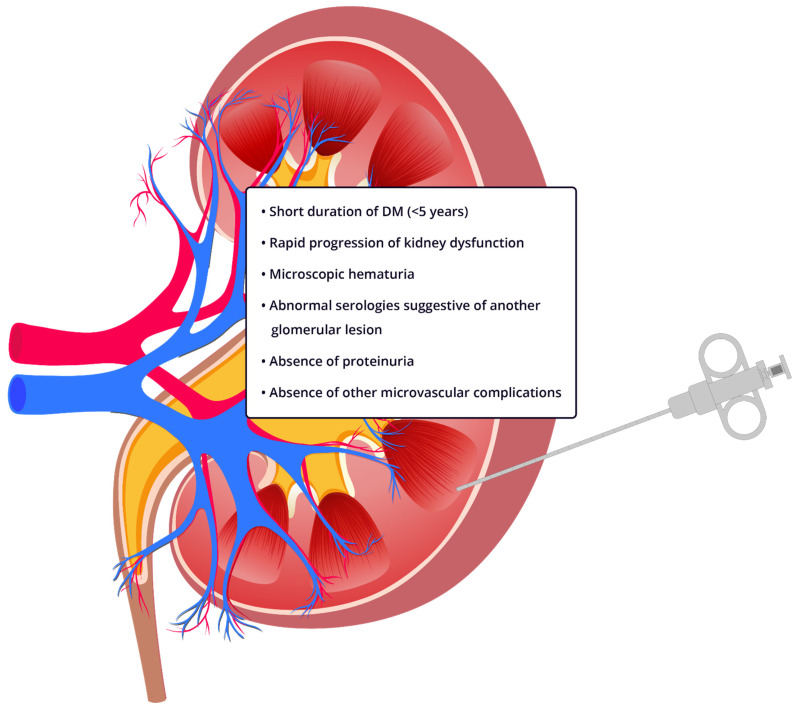
Indications for kidney biopsy in patients with diabetes and suspected nondiabetic kidney disease. One or more of the following criteria should raise suspicion of NDKD and prompt a kidney biopsy in diabetic patients.

Diabetic retinopathy is associated with diabetic glomerulosclerosis according to data from a meta-analysis including 26 studies (OR 5.7, 95% IC 3.45–9.34) [53] and confirmed by contemporary studies [54], with a sensitivity of 87% and specificity of 93% [55]. Duration of diabetes < 5 years was predictive of NDKD (75% sensitivity and 70% specificity) across multiple studies [5,6,55]. The absence of diabetic retinopathy, a duration of diabetes of less than 5 years, or an atypical course of DKD (e.g., rapid rise in proteinuria and/or decline in GFR) should raise suspicion of NDKD [5] and lead to a kidney biopsy (Figure 2). Performing the kidney biopsy in earlier stages of CKD in diabetic patients could also improve the outcomes of this group of patients, as most biopsies are performed in late stages [6]. There is an emerging use of kidney tissue in biomarker discovery in DKD [56]. The feasibility and safety of obtaining research tissue during clinically indicated kidney biopsies would help to enhance the use for future trials. Successful procurement of research cores has been reported in 89% of participants for the TRIDENT study [57] and is a staple of the landmark Kidney Precision Medicine Project sponsored by the National Institutes of Health.

## 6. Conclusions

In patients with type 2 DM, NDKD could represent a frequent, treatable pathologic finding. In cases manifesting an atypical course of DKD, NDKD alone or superimposed on diabetic glomerulosclerosis may be present in up to two-thirds of all cases. The true prevalence of NDKD is unknown and different across regions, influenced by background population (racial ethnicity and rates of obesity) and biased by kidney biopsy policies and the retrospective nature of most studies. The majority of kidney biopsies performed in patients with diabetes occur at late stages of disease, meaning that NDKD is often diagnosed late when glomerular disease is severely advanced. A more extensive use of kidney biopsies in patients with DM in clinical trials can bring a better understanding of NDKD.

Diabetic patients with glomerular disease represent an important patient population that has been excluded or under-enrolled in most clinical trials investigating management of glomerular diseases [5], limiting evidence to guide management. New therapeutic options that do not involve immunosuppression are available and can delay progression in DM patients with DKD or nondiabetic glomerular diseases. It has been estimated that NDKD is responsible for a significant percentage of cases that reach ESKD. In patients with type 2 diabetes, NDKD could represent a potentially reversible cause of kidney damage which merits greater attention from the scientific community.
